# IgG N-glycome changes during the course of severe COVID-19: An observational study

**DOI:** 10.1016/j.ebiom.2022.104101

**Published:** 2022-06-27

**Authors:** Tea Petrović, Amrita Vijay, Frano Vučković, Irena Trbojević-Akmačić, Benjamin J. Ollivere, Damir Marjanović, Tamer Bego, Besim Prnjavorac, Lovorka Đerek, Alemka Markotić, Ivica Lukšić, Ivana Jurin, Ana M. Valdes, Irzal Hadžibegović, Gordan Lauc

**Affiliations:** a Genos Ltd, Glycoscience Research Laboratory, Zagreb, Croatia; b Injury, Inflammation and Recovery Unit, School of Medicine, University of Nottingham, Nottingham NG7 2UH, United Kingdom; c Faculty of Pharmacy and Biochemistry, University of Zagreb, Croatia; d International Burch University, Sarajevo, Bosnia and Herzegovina; e Center for Applied Bioanthropology, Institute for Anthropological Research, Zagreb, Croatia; f University of Sarajevo, Faculty of Pharmacy, Department of Pharmaceutical biochemistry and Laboratory diagnostics, Sarajevo, Bosnia and Herzegovina; g General Hospital Tešanj, Bosnia and Herzegovina; h University of Zagreb School of Medicine, Dubrava University Hospital, Zagreb, Croatia; i University Hospital for infectious diseases “Fran Mihaljević”, Zagreb, Croatia; j Faculty of Medicine of the University of Rijeka, Rijeka, Croatia; k Faculty of Medicine, Catholic University of Croatia, Zagreb, Croatia

**Keywords:** Bisecting GlcNAc, Galactosylation, COVID-19, Molecular epidemiology, IgG glycosylation, SARS-CoV-2

## Abstract

**Background:**

The severe acute respiratory syndrome coronavirus-2 (SARS-CoV-2) causes a respiratory illness named coronavirus disease 2019 (COVID-19), which is one of the main global health problems since 2019. Glycans attached to the Fc portion of immunoglobulin G (IgG) are important modulators of IgG effector functions. Fc region binds to different receptors on the surface of various immune cells, dictating the type of immune response. Here, we performed a large longitudinal study to determine whether the severity and duration of COVID-19 are associated with altered IgG glycosylation.

**Methods:**

Using ultra-high-performance liquid chromatography analysis of released glycans, we analysed the composition of the total IgG N-glycome longitudinally during COVID-19 from four independent cohorts. We analysed 77 severe COVID-19 cases from the HR1 cohort (74% males, median age 72, age IQR 25-80); 31 severe cases in the HR2 cohort (77% males, median age 64, age IQR 41-86), 18 mild COVID-19 cases from the UK cohort (17% males, median age 50, age IQR 26-71) and 28 mild cases from the BiH cohort (71% males, median age 60, age IQR 12-78).

**Findings:**

Multiple statistically significant changes in IgG glycome composition were observed during severe COVID-19. The most statistically significant changes included increased agalactosylation of IgG (meta-analysis 95% CI [0.03, 0.07], adjusted meta-analysis P= <0.0001), which regulates proinflammatory actions of IgG via complement system activation and indirectly as a lack of sialylation and decreased presence of bisecting *N*-acetylglucosamine on IgG (meta-analysis 95% CI [-0.11, -0.08], adjusted meta-analysis P= <0.0001), which indirectly affects antibody-dependent cell-mediated cytotoxicity. On the contrary, no statistically significant changes in IgG glycome composition were observed in patients with mild COVID-19.

**Interpretation:**

The IgG glycome in severe COVID-19 patients is statistically significantly altered in a way that it indicates decreased immunosuppressive action of circulating immunoglobulins. The magnitude of observed changes is associated with the severity of the disease, indicating that aberrant IgG glycome composition or changes in IgG glycosylation may be an important molecular mechanism in COVID-19.

**Funding:**

This work has been supported in part by Croatian Science Foundation under the project IP-CORONA-2020-04-2052 and Croatian National Centre of Competence in Molecular Diagnostics (The European Structural and Investment Funds grant #KK.01.2.2.03.0006), by the UKRI/MRC (Cov-0331 - MR/V027883/1) and by the National Institutes for Health Research Nottingham Biomedical Research Centre and by Ministry Of Science, Higher Education and Youth Of Canton Sarajevo, grant number 27-02-11-4375-10/21.

**Research in context**

**Evidence before the study**

The rapid global spread of severe acute respiratory syndrome coronavirus 2 (SARS-CoV-2) caused coronavirus disease 2019 (COVID -19) and brought enormous pressure and catastrophic consequences to public health and medical systems worldwide. Most infected individuals recovered from infection without symptoms or with mild fever and cough, while some cases are characterized by dyspnea, cytokine storm, respiratory failure, and death. The varying severity of disease symptoms is one of the key issues in COVID -19 pandemics. Immunoglobulin G (IgG), the most abundant glycoprotein in human blood plasma, is one of the key molecules in the immune response. Glycosylation of IgG has been poorly studied in various severity of COVID -19. Most studies featured limited sample numbers, and current literature has been focused on afucosylation, anti-S IgG1 glycosylation.

**Added value of the study**

We performed a large longitudinal observational study to determine whether severity and duration of COVID-19 are associated with altered IgG glycosylation. We have analysed the composition of total IgG N-glycome in patients with severe, mild, or asymptomatic COVID-19 from four independent cohorts. We made considerable efforts to identify patients in whom acute COVID -19 was the main disease, so we excluded all patients with other severe concomitant diseases. Longitudinal samples were obtained from all patients, thus identifying the dynamics of changes in each individual patient. Our results revealed that the magnitude of the observed changes was related to the severity of the disease, suggesting that aberrant changes in IgG glycosylation may be an important molecular mechanism in COVID-19.

**Implications of all the available evidence**

In severe COVID-19 patients, IgG glycome was altered in a manner that makes IgG more pro-inflammatory, indicating that loss of immunosuppressive effect of IgG may have a role in disease severity. Furthermore, this is the first demonstration that acute viral infection can cause rapid and extensive changes in the total IgG glycome.

Alt-text: Unlabelled box

## Introduction

The severe acute respiratory syndrome-related coronavirus-2 (SARS-CoV-2) is an RNA virus that has caused the coronavirus disease 2019 (COVID-19) pandemic. SARS-CoV-2 belongs to Coronaviridae family and represents seventh coronavirus (CoV) known to infect humans.^1^ To date, SARS-CoV-2 has infected over 397 million people and caused more than 5.7 million deaths.^2^ Clinical features like low levels of neutralizing antibodies against SARS-CoV-2^3^ and prolonged disease in some patients indicate that SARS-CoV-2 can evade human immune surveillance more effectively than SARS-CoV-1.^4^ SARS-CoV-2 entry into host cells is an essential step of viral infectivity and pathogenesis, mediated by the spike glycoprotein S interaction, predominantly with angiotensin-converting enzyme 2 (ACE2).^5^ After SARS-CoV-2 binding to a cell surface receptor, receptor transmembrane protease serine 2 (TMPSRR2) cleaves SARS-CoV-2 S glycoprotein between S1 and S2 domains, enabling S1 detachment, conformational changes of S2, and fusion of viral and host cell membrane.^6^ Alternatively, SARS-CoV-2 can utilize the endosomal pathway to enter the cells.^7^ These features of SARS-CoV-2 entry contribute to its rapid spread within human population,^3^^,^^5^^,^^7^ with symptoms ranging from asymptomatic to severe, even life-threatening conditions, characterized by high levels of inflammatory cytokines, alveolar inflammatory infiltrates, and vascular microthrombi formation.^8^

Glycosylation mediates several cellular functions and glycans represent one of the main molecular tools against pathogens. Glycosylation changes can both modulate inflammatory responses and allow the virus to escape the immune system. As such, glycans have emerged as new biomarker candidates for COVID-19. Immunoglobulin G (IgG) is an important effector molecule of the immune system. Glycosylation of IgG has been shown to differ between mild and severe COVID-19 cases, as well as between severe COVID-19 cases and healthy controls.^9^ IgG consists of two functionally distinct parts, antigen-binding (Fab) and fragment crystallizable (Fc). The Fc region binds to different receptors on the surface of various immune cells, dictating the type of immune response. Fc receptors for IgG expressed on macrophages and natural killer (NK) cells are involved in multiple immune processes, such as complement-dependent cytotoxicity (CDC), and antibody-depended cell-mediated cytotoxicity (ADCC).

Recent studies on IgG glycome show that SARS-CoV-2 infection is associated with the decrease in fucosylation of IgG and a low level of IgG sialylation, which triggers ADCC-branch of the immune response and contributes to the elevation of inflammatory cytokines.^9^ Also, our previous study on total IgG shows that changes in IgG glycome composition are closely related to the loss of the immunosuppressive function and contribute to the immune-mediated pathologies of SARS-CoV-2 infection.^10^ Chakraborty and colleagues show that disease severity in COVID-19 correlates with the presence of proinflammatory IgG Fc structures, including afucosylated anti-RBD IgG1.^11^ Furthermore, they also showed that early non-neutralizing, afucosylated IgG1 antibodies specific to SARS-CoV-2 were associated with progression from mild to more severe COVID-19.^12^ Another study by Larsen and colleagues showed that afucosylated antigen-specific IgG may be an important element in the defence against SARS-CoV-2.^13^ Also, they claimed that excessive afucosylated IgG response in SARS-CoV-2 may promote the exacerbation of COVID-19, by producing pro-inflammatory cytokines IL-6 and IL-8.^13^ A novel study by Hoepel and colleagues showed that high anti-spike IgG titers from patients with severe COVID-19 induce excessive inflammatory response because of their altered glycosylation, particularly low fucosylation.^14^

The analysis of IgG glycans has shown that the change in their composition associates with various inflammatory and autoimmune diseases, and it has been recently discovered that they are a good biomarker of biological and chronological age.^15^^,^^16^ Some studies suggest that biological age, rather than chronological age, of affected patients, might be the critical factor to systematically assess COVID-19.^17^^,^^18^

Therefore, it is not surprising that IgG glycosylation has been studied in COVID-19 infection as one of the factors influencing COVID-19 severity.^10^, ^11^, ^12^, ^13^ However, mild, and asymptomatic COVID-19 cases, as well as longitudinal changes during COVID-19, are still underexplored. Here, we analysed IgG N-glycome composition from four independent cohorts: 77 and 31 patients with severe COVID-19 from Zagreb, Croatia (HR1 and HR2, respectively), 18 individuals with mild or asymptomatic COVID-19 from hospitals in Nottingham, United Kingdom (UK), and 28 patients with mild COVID-19 from Tešanj, Bosnia and Hercegovina (BiH) were analysed.

## Methods

### Participants

Biological samples were obtained from 108 patients with severe and 46 patients with mild COVID -19 from four independent cohorts. From that, 77 patients (57 males and 20 females) aged 25-80 and 31 (24 male and 7 females) aged 41-86 with PCR confirmed SARS-CoV-2 infection from the University Hospital Dubrava, Croatia (HR1 and HR2, respectively), 18 individuals positive for SARS-CoV-2 antibodies (3 males and 15 females) aged 25-80 from Injury, Inflammation and Recovery Unit, School of Medicine, University of Nottingham, Nottingham (UK), 28 (20 male and 8 female) aged 12-78 patients from General Hospital Tešanj, Bosnia and Herzegovina (BiH) were included. The blood samples for IgG isolation were collected in multiple time points weekly within two months (UK) or multiple times during hospitalization (BiH, HR1, HR2). University Hospital Dubrava was organized as a dedicated SARS-CoV-2 hospital from November 2020 to June 2021 and samples were collected during two subsequent waves of the pandemics (HR1 cohort from November to December 2020, and HR2 cohort from March to April 2021). Together with the clinical samples collection, patient data has also been recorded and entered into internal database of each clinical centre. Before glycan analysis, patient info has been anonymised by introducing a unique patient ID which was used as an identifier during laboratory glycan analysis.

### Study design

Using the developed high-throughput UHPLC approach for IgG glycosylation analysis, we analysed IgG glycome composition in 108 patients with severe and 46 patients with mild COVID -19 from four independent cohorts. For the UK cohort samples were collected weekly within two months. For BiH, HR1 and HR2 after obtaining informed consent from patients, samples were collected during hospital admission. Regarding severity, patients in this study were divided into several groups (Table 1). For the UK cohort we have only patients with asymptomatic and mild disease, for BiH cohort we have patients with mild and moderate disease, and we combined them into a single group. For HR1 and HR2 we have only patients with severe or critical disease which were merged into a single group. To make the cohorts more uniform in terms of longitudinal sample collection and minimize potential biases, we have excluded patients that were already seropositive at the time of recruitment in the UK cohort and those that had other comorbidities in HR1 and HR2 cohorts before glycan analysis. This is an observational study and samples were collected as samples of convenience. No statistical calculation of sample size or sensitivity data was performed; sample size was determined based on availability.

**Table 1:** Severity of patients included in each cohort analysed in this study.

Levels of severity	Description	Cohort			
		UK	BiH	HR1	HR2
Asymptomatic	Individuals seropositive for COVID-19 but without any symptoms of COVID-19	X			
Mild	Individuals with no evidence of pneumonia, but with typical clinical manifestations	X	X		
Moderate	Individuals with evidence of pneumonia, however without need of invasive mechanical ventilation		X		
Severe	Individuals with need of hospital intensive care unit and need of invasive mechanical ventilation			X	X
Critical	Individuals with need of immediate invasive mechanical ventilation and admission to hospital intensive care unit or need for extracorporeal circulation or deceased during the hospitalization			X	X

### Sample preparation

#### Isolation of IgG from human plasma

IgG was isolated using a 96-well protein G monolithic plate (BIA Separations, Slovenia, Cat No. 120.1012-2)^19^ using a protocol described by Trbojević-Akmačić and colleagues.^20^ After IgG isolation, IgG eluates were heated at 65°C for 30 minutes to reduce the risk of any potential residual virus in the IgG eluate. An appropriate volume of IgG was aliquoted in a PCR plate (Thermo Scientific, UK, Cat. No. AB1300) and dried in a vacuum centrifuge, if needed. Deglycosylation, released N-glycan labeling and clean-up were performed using two different kits, GlycoWorks RapiFluor-MS N-Glycan Kit (Waters, USA, Cat. No. 176003910) and AdvanceBio Gly-X N-glycan Prep with InstantPC Kit (Agilent, USA, Cat. No. GX96-IPC) according to the manufacturer's instructions.

#### GlycoWorks RapiFluor-MS N-Glycan Kit

For samples collected in Tešanj, BiH and Zagreb, HR GlycoWorks RapiFluor-MS N-Glycan Kit was used. The protocol for this analysis was described in detail by Deriš and colleagues.^21^ Briefly, dried IgG eluate (average mass of 15 µg) was resuspended in ultrapure water and 5 % GlycoWorks RapiGest SF solution (Waters, USA)was added to each sample to denature IgG. N-glycans from samples were enzymatically released from IgG by GlycoWorks Rapid PNGase F (Waters, USA) and labelled with GlycoWorks RapiFluor-MS Solution (Waters, USA). After labelling, acetonitrile (ACN, Honeywell, USA, Cat. No. 34967) was added to the samples, which were then immediately transferred to a GlycoWorks HILIC µElution Plate (Waters, USA) prior to clean up procedure by hydrophilic interaction liquid chromatography solid-phase extraction (HILIC-SPE). Glycans were eluted with GlycoWorks SPE Elution Buffer, 200 mmol/L ammonium acetate/ACN (95:5, v/v) pH 7 (Waters, USA), and diluted with 310 µL of GlycoWorks Sample Diluent, DMF/ACN (32:68, v/v) (Waters, USA). The 40 µL of each sample was transferred to vials for ultra-high-performance liquid chromatography based on hydrophilic interactions (HILIC-UHPLC) analysis, while the remainder of the samples were stored at -20 °C.

### AdvanceBio Gly-X Kit

For samples collected in Nottingham, UK, AdvanceBio Gly-X N-glycan Prep with InstantPC Kit was used (Agilent, USA, Cat. No. GX96-IPC). An appropriate volume of eluate containing IgG (∼2 mg/mL) was aliquoted in a PCR plate (Agilent, USA, Cat. No. GX96-100). Reagents and buffers used for this analysis were in detail described in the protocol.^22^ Gly-X Denaturant (Agilent, USA, Cat. No. GX96-100) and samples were added on Gly-X Deglycosylation Plate (Agilent, USA, Cat. No. GX96-100). The plate containing the samples was incubated at 99 °C for 3 minutes, after which it was left at room temperature to cool down for 2 minutes. For deglycosylation, the volume of 2 µL of N-glycanase Working Solution (Agilent, USA, Cat. No. GX96-100) was added to each sample and incubated at 50 °C for 5 minutes. The released N-glycans were labelled with InstantPC Dye Solution (Agilent, USA, Cat. No. GX96-101). To each N-glycan sample, 5 µL of the label was added, and the samples were incubated at 50 °C for 1 minute. To precondition the Gly-X Cleanup Plate (Agilent, USA, Cat. No. GX96-102) 400 µL of Load/Wash Solution was added to each well. Load/Wash Solution was added to samples, which were then immediately transferred to Gly-X Cleanup Plate prior to clean up procedure by HILIC-SPE. The samples were eluted with 100 µL of Gly-X InstantPC Eluent (160 mM Ammonium Formate w/10% (v/v) ACN, Agilent, USA, Cat. No. GX96-102), and fractions were collected into the 0.8 mL collection plate.

### HILIC-UHPLC analysis

Labelled and purified IgG *N*-glycans were analysed on Waters Acquity UHPLC instrument (Milford, MA, USA) consisting of a quaternary solvent manager, sample manager and a fluorescence detector. The separation temperature was 60°C, and samples were maintained at 10°C before injection.

RapiFluor-MS labelled glycans were separated on a Waters Glycan Premier BEH Amide chromatography column, 100 × 2.1 mm i.d., 1.7 μm BEH particles (Waters, USA, Cat. No. 186009523), with solvent A (50 mM ammonium formate, pH 4,4) and ACN as solvent B. Separation method used a linear gradient of 75–61.5% ACN (Honeywell, USA) (v/v) at a flow rate of 0.4 ml/min in a 42-minute analytical run. Excitation and emission wavelengths were set to 256 nm and 425 nm, respectively. Obtained chromatograms were separated into 22 peaks for which the glycan structures were described by Keser and colleagues^23^ (Supp Table 2).

InstantPC labelled glycans were separated on an Agilent AdvanceBio Glycan Mapping amide HILIC chromatography column, 150 × 2.1 mm i.d., 1.8 μm particle size (Agilent, USA, Cat. No. 859700-913), with solvent A (100 mM ammonium formate, pH 4,4) and ACN (Honeywell, USA) as solvent B. Separation method used a linear gradient of 78-63,7% ACN (v/v) at a flow rate of 0.5 ml/min in a 30-minute analytical run. The chromatograms were separated into 25 chromatographic peaks (Supp Table 2) and glycan structures annotated as described in the following subsection.

To remove experimental variation from measurements, normalization and batch correction were performed on high-throughput UHPLC data.^24^ Normalization by total area was performed where the amount of *N*-glycans in each chromatographic peak was expressed as a percentage of total integrated area (% Area). Prior to batch correction, normalized glycan measurements were log-transformed due to right-skewness of their distributions and multiplicative nature of batch effects. Batch correction was performed on log-transformed measurements using ComBat method (R package sva),^25^ where technical source of variation (which sample was analysed on which plate) was modelled as batch covariate. To get measurements corrected for experimental noise, estimated batch effects were subtracted from log-transformed measurements. From directly measured glycan peaks we calculated derived traits which average glycosylation traits such as G0 – glycans without galactose, G1 – glycans with one galactose, G2 – glycans with two galactoses, S – percentage of all glycans with sialic acid, F –fucosylated glycans, and B –glycans with bisecting *N*-acetylglucosamine (GlcNAc) across different individual glycan structures (Supp Table 2).

### Structural characterization of N-glycans

For structural characterization of IgG N-glycans prepared with AdvanceBio Gly-X N-glycan Prep with InstantPC Kit, the released and labelled N-glycans were analysed by liquid chromatography-mass spectrometry (LC-MS) on a BioAccord LC-MS System (Waters, USA). Chromatographic conditions were the same as described above for the analysis of InstantPC labelled glycans. The RDa mass detector was used in-line via electrospray ionization in positive mode. The settings were as follows: scan range, 50–2000 m/z; capillary voltage, 1.5 kV; cone voltage, 45 V; desolvation temperature, 300°C; and sampling rate, 2 Hz. Acquired data were automatically processed using the UNIFI 1.9.9.3 Scientific Information System. For the glycan identification, MS1 sum spectra were generated around the retention times (RT) of the chromatographic peak and annotation of MS1 sum spectra were inferred from the m/z values and deduced using GlycoMod software (https://web.expasy.org/glycomod/, accessed on the 7^th^ of July 2021)^26^ MS interpretation was based on previously reported structures^19^ and biosynthetic pathways.

### Statistical analysis

Longitudinal analysis of patient samples through their observation period was performed by implementing a maximum likelihood (ML)‐based linear mixed-effects model. Analyses included glycan measurement as dependent continuous variable, time (days) was included both as fixed continuous effect and random slope while individual ID was included in a model as a random intercept (Glycan ∼ Time + (Time|PatientID)). An unstructured covariance structure was used to model the within‐patient errors. To draw inferences on fixed effects of the model, likelihood ratio test was used. LMM analyses were implemented using lme4 package (lmer(method=“ML”)). For all successfully analysed samples complete glycan and clinical data was available, and no exclusion/imputation procedure was performed. LMM analyses were performed for each cohort separately and the results were aggregated using random-effects meta-analysis approach (two-stage individual patient data (IPD) meta-analysis). Two separate meta-analyses were performed – one using UK and BiH results (mild COVID), and another one using HR1 and HR2 results (severe COVID). Maximum Likelihood method was used to estimate τ^2. I^2 and τ^2 were reported as measures of heterogeneity. Meta-analyses were implemented using metagen package (metagen(method = “ML”). Prior to analyses, glycan variables were all transformed to standard Normal distribution (mean=0, sd=1) by inverse transformation of ranks to Normality (R package "GenABEL", function rntransform). Using rank transformed variables in analyses makes estimated effects of different glycans in different cohorts comparable as transformed glycan variables have the same standardized variance. False discovery rate was controlled using Benjamini-Hochberg procedure (function p.adjust(method = “BH”)). Multiplicity adjustment was applied within subgroups – one adjustment for mild COVID analysis, and another for severe COVID analysis. Data was analysed and visualized using R programming language (version 3.0.1).

### Ethics

Biological samples were obtained from hospitals in Nottingham, United Kingdom (UK), Tešanj, Bosnia and Hercegovina (BiH), and Zagreb, Croatia (HR). The study protocol conformed to the ethical guidelines of the 1975 Declaration of Helsinki and the study was approved by ethical committees of the Faculty of Medicine & Health Sciences Research Ethics Committee, Nottingham University Hospital (Reference NO: FMHS 41-0620), Ethics Committee of Hospital in Tešanj, Bosna and Hercegovina (Reference NO: 01-4-18721) and Ethics Committee of University Hospital Dubrava, Croatia (Reference NO: 2020/2409-10). All participants gave informed consent.

### Role of funding source

The research was funded by Croatian Science Foundation under the project IP-CORONA-2020-04-2052 and Croatian National Centre of Competence in Molecular Diagnostics (The European Structural and Investment Funds grant #KK.01.2.2.03.0006), by the UKRI/MRC (Cov-0331 - MR/V027883/1) and by the National Institutes for Health Research Nottingham Biomedical Research Centre and by Ministry of Science, Higher Education and Youth of Canton Sarajevo, grant number 27-02-11-4375-10/21. The funding sources had no role in study design, data collection, data analyses, interpretation, or writing of the manuscript.

## Results

IgG N-glycome composition (combined Fc and Fab glycans) was analysed in 505 samples collected in multiple time points from 108 severe patients and 46 patients with a mild form of COVID-19 from four independent cohorts (Table 2). The descriptive information on mild and severe COVID-19 patients are presented in Table 2. Total IgG N-glycome composition was determined by UHPLC analysis of glycans labelled with RapiFluor-MS or InstantPC as described in the Materials and methods section. From the individually quantified chromatographic peaks derived glycan traits were calculated to represent a portion of structurally similar glycan structures and the statistical analysis was performed on these main summary features of the IgG N-glycome composition (Supp Table 3).

**Table 2:** Descriptive information about COVID-19 patients included in the study.

	HR1 (n=77)	HR2 (n=31)	UK (n=18)	BiH (n=28)
Disease severity	Severe	Severe	Mild	Mild
Sex (Male/Female)	57/20	24/7	3/15	20/8
Age (Median [IQR])	72 years (25-80)	64 years (41-86)	50 years (26-71)	60 years (12-78)
Ethnicity data	77 Caucasians	31 Caucasians	15 Caucasians / 3 B.M. (Black, Asian and minority ethnic)	28 Caucasians

**Figure 1:**
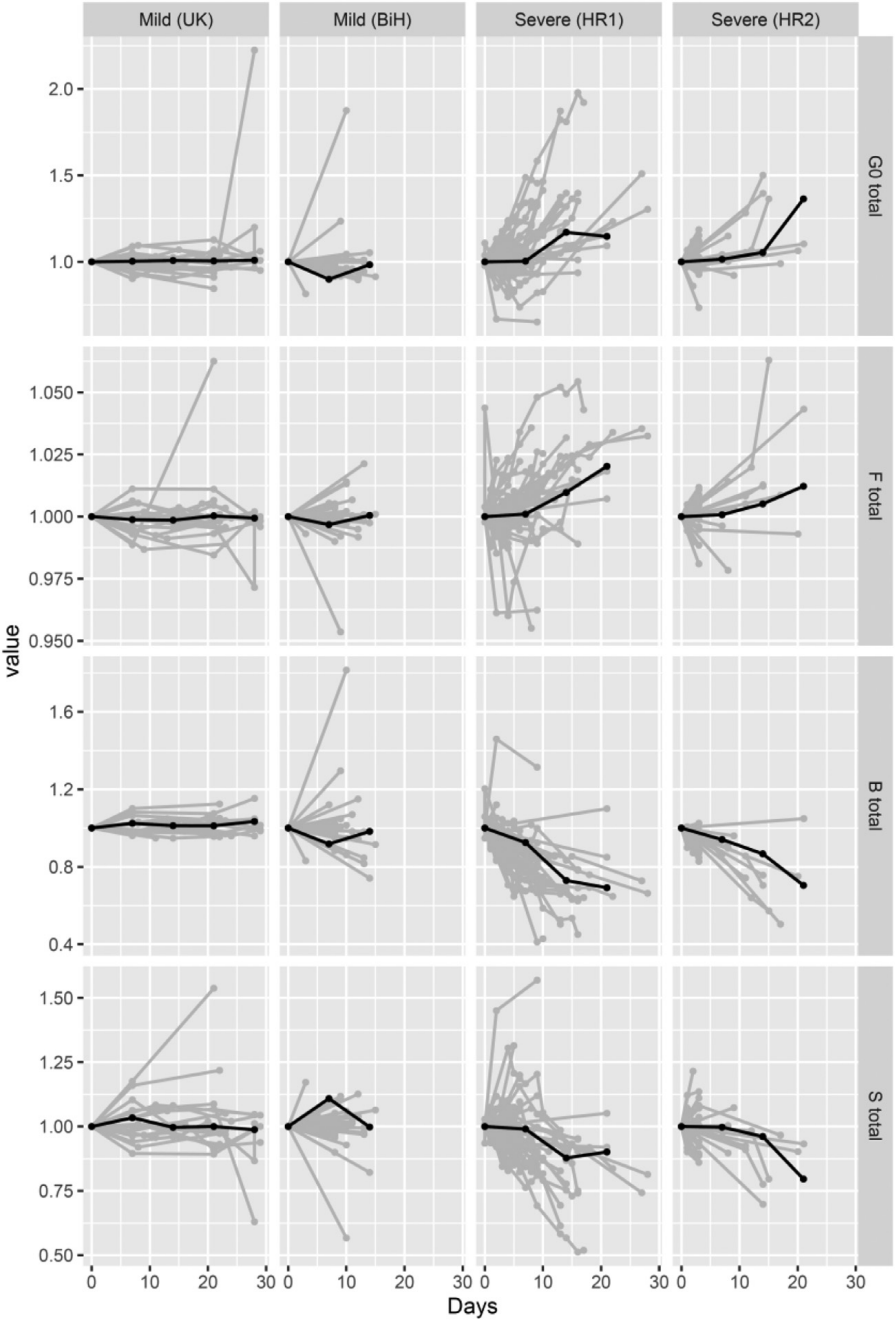
Alterations in IgG glycome composition during mild (UK, n= 18; BiH, n=28) and severe (HR1, n=77; HR2, n=31) COVID-19.

**Figure 2:**
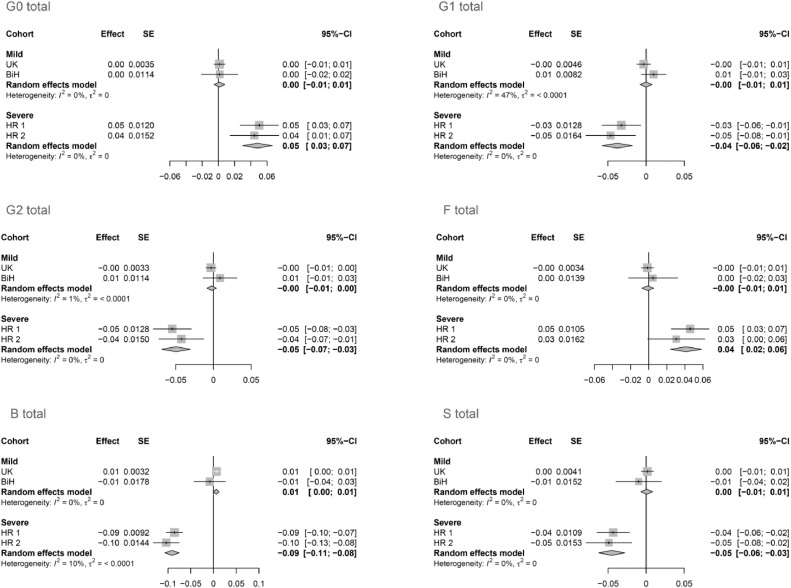
Effects of mild (UK, n= 18; BiH, n=28) and severe (HR1, n=77; HR2, n=31) COVID-19 on the IgG glycome. Results of meta-analysis are presented. SE – standard error; 95% CI – 95% confidence interval; G0 – agalactosylated N-glycans, G1 – N-glycans with one galactose, G2 – N-glycans with two galactoses, S – sialylated N-glycans, B – N-glycans with bisecting GlcNAc, F – N-glycans with core fucose.

Meta-analysis of effects of COVID-19 was performed for the two cohorts of hospitalized patients from Croatia, and separately for a cohort of asymptomatic/mild cases from UK and BiH (Table 2, Figures 1, 2, Supp Figure 3). Statistically significant changes in the IgG glycome composition were observed during severe COVID-19. The most extensive changes in severe COVID-19 were observed in the level of bisecting GlcNAc (meta-analysis 95% CI [-0.11, -0.08], adjusted meta-analysis P= <0.0001, Table 3, Figures 1, 2). In patients with asymptomatic/mild COVID-19 no statistically significant changes in bisecting GlcNAc were observed.

**Table 3:** Statistical analysis of IgG glycome composition changes during severe and mild COVID-19.

Glycan	HR 1_ effect	HR 1_ 95% CI	HR 1 _ pval	HR 2_ effect	HR 2_ 95%CI	HR 2_ pval	Meta_ effect	Meta_ 95% CI	Meta_ p.val	Meta_ p.adj
G0 total	0·0504	(0·027-0·074)	0·0002	0·0443	(0·014-0·074)	0·0153	0·0481	(0·030-0·067)	<0·0001	<0·0001
G1 total	-0·0324	(-0·058- -0·001)	0·0196	-0·0464	(-0·079–0·014)	0·0111	-0·0377	(-0·057–0·018)	0·0002	0·0002
G2 total	-0·0546	(-0·080–0·030)	0·0001	-0·0426	(-0·072–0·013)	0·0287	-0·0496	(-0·069–0·031)	<0·0001	<0·0001
F total	0·0459	(0·025-0·067)	0·0002	0·0306	(-0·001-0·062)	0·0951	0·0413	(0·024-0·059)	<0·0001	<0·0001
B total	-0·0858	(-0·086–0·068)	<0·0001	-0·1039	(-0·132–0·075)	<0·0001	-0·0912	(-0·107–0·073)	<0·0001	<0·0001
S total	-0·0434	(-0·064–0·022)	0·0005	-0·0490	(-0·080–0·020)	0·0127	-0·0452	(-0·063–0·028)	<0·0001	<0·0001

Glycan	UK _ effect	UK _ 95% CI	UK _ pval	BiH_ effect	BiH_ 95% CI	BiH_ pval	Meta_ effect	Meta_ 95% CI	meta_ p.val	meta_ p.adj
G0 total	0·0013	(-0·005-0·008)	0·7276	0·0015	(-0·021-0·024)	0·8972	0·0012	(-0·005-0·008)	0·7009	0·9840
G1 total	-0·0032	(-0·012-0·006)	0·5034	0·0098	(-0·006-0·026)	0·2427	-0·0001	(-0·008-0·008)	0·9840	0·9840
G2 total	-0·0003	(-0·009-0·003)	0·3280	0·0086	(-0·014-0·031)	0·4592	-0·0025	(-0·009-0·004)	0·4394	0·9840
F total	-0·0014	(-0·008-0·005)	0·6988	0·0048	(-0·022-0·032)	0·7208	-0·0010	(-0·008-0·006)	0·7628	0·9840
B total	0·0066	(0·000-0·012)	0·0448	-0·0083	(-0·045-0·026)	0·6463	0·0061	(0·000-0·012)	0·0494	0·2963
S total	0·0015	(-0·006-0·009)	0·7130	-0·0099	(-0·040-0·020)	0·516	0·0007	(0·001-0·008)	0·8512	0·9840

G0 – agalactosylated N-glycans, G1 – N-glycans with one galactose, G2 – N-glycans with two galactoses, S – sialylated N-glycans, B – N-glycans with bisecting GlcNAc, F – N-glycans with core-fucose; CI- Confidence interval.

*Adjustment for multiple testing using Benjamini-Hochberg procedure.

Statistically significant changes in the level of galactosylation were also observed in severe COVID-19. Glycans without galactose (G0) increased (meta-analysis 95% CI [0.03, 0.07], adjusted meta-analysis P= <0.0001, Table 3, Figure 1), while glycans with one (G1) or two (G2) galactoses decreased during severe COVID-19 (for G1 meta-analysis 95% CI [-0.06, -0.02], adjusted meta-analysis P= 0.0002, and for G2 meta-analysis 95% CI [-0.07, -0,03], adjusted meta-analysis P= <0.0001 Table 3, Supp Figure 1). During asymptomatic/mild COVID-19 IgG galactosylation did not statistically significantly change. Fucosylation also increased during severe COVID-19 (meta-analysis 95% CI [0.02, 0.06], adjusted meta-analysis P= <0.0001, Table 3, Figure 1), while levels of sialylated IgG N-glycan structures decreased (meta-analysis 95% CI [-0.06, -0.03], adjusted meta-analysis P= <0.0001, Table 3, Figure 1).

Standardised glycan measurements are represented on the y-axis, while time in days is presented on the x-axis. Black dots represent 7-day, cohort-specific averages of standardized glycan measurements. G0 – agalactosylated N-glycans, S – sialylated N-glycans, B – N-glycans with bisecting GlcNAc, F – N-glycans with core fucose. Follow-up data for 30 days is presented. Additional information is available in Table 3.

## Discussion

This study represents the first comprehensive longitudinal analysis of IgG N-glycosylation in COVID-19 of different severity. To investigate whether COVID-19 itself triggers a statistically significant change in the IgG glycome composition, we performed a longitudinal analysis of IgG N-glycome in sera drawn at different time points. By analysing 108 severe COVID-19 patients and 46 mild COVID-19 patients from four independent cohorts we demonstrated that COVID-19 severity associates with statistically significant alterations in IgG glycome composition. The increase in IgG fucosylation, and the decrease in IgG galactosylation, bisecting GlcNAc and sialylated glycans were observed. The effects of COVID-19 on the IgG glycome in both cohorts of the meta-analysis were in the same direction, which allowed replication of the main findings.

The level of bisecting GlcNAc was the most prominent feature distinguishing severe and mild COVID-19 in all analysed cohorts. Decreased bisection was found in severe patients. Higher levels of bisecting GlcNAc on IgG were reported to indirectly affect affinity for FcγRs and enhance ADCC by inhibiting fucoslyation.^27^ Recent reports suggest that severe COVID-19 patients present low levels of bisection both on total IgG N-glycome^10^ as well as on anti-S and anti-N IgG1 compared to mild cases.^13^ In contrast, a novel study by Pongracz and colleagues found a rapid increase in total IgG1 in bisection within days and weeks after the onset of the disease.^28^ Moreover Chakraborty and colleagues found no difference in anti-RBD IgG1 bisection between ICU and non-ICU patients.^11^ Differences in the results of these studies could be primarily attributed to differences in the studied analytes, while here we analysed glycosylation on total plasma IgG, Pongracz and colleagues^28^ and Chakraborty and colleagues^29^ exclusively studied anti-S and total IgG1, and anti-RBD IgG1, respectively. Moreover, Larsen and colleagues^13^ report differences in glycosylation between anti-S and anti-N IgG1, while Pongacz and colleagues^28^ stress out that days since COVID-19 onset is one of the major confounders of anti-S IgG1 glycosylation. All these results, although seemingly controversial, support the hypothesis of the highly dynamic nature of IgG glycosylation and immune system response during severe COVID-19, as well as the potential existence of some predisposing factors for more severe COVID-19.

The same study by Pongracz and colleagues reported low fucosylation on anti-S as compared to total IgG1 at hospitalization, but no difference in fucosylation between hospitalized ICU patients and hospitalized non-ICU patients.^28^ Likewise, other studies showed that lower antigen-specific IgG fucosylation is associated with the immune response to enveloped viruses and viral infection severity, while total IgG fucosylation is relatively stable.^11^^,^^13^ In our study we observed that the total IgG fucosylation increases during severe COVID-19, which confirms previous results.^13^ Hou and colleagues reported lower levels of total IgG fucosylation in severe COVID-19 cases compared with healthy controls, but higher levels of total IgG fucosylation in severe COVID-19 cases compared with mild cases.^9^ As mentioned above, these reported differences in fucosylation are most likely to some extent a reflection of specific glycosylation profiles of different antigen-specific or total IgG that have been analysed in each study, as well as dynamics of antigen-specific IgG presence during the course of COVID-19. This dynamic immune response and specific glycosylation profile of antigen specific IgGs may or may not be observed on the level of total IgG depending on their concentration as well as time of sampling. Alternatively, these differences in IgG fucosylation may be associated with different molecular mechanisms involved in the immune response to SARS-CoV-2. Truly, increased levels of pro-inflammatory cytokines are not present in all severe patients.^30^ Massive release of pro-inflammatory cytokines including the type-I interferons (IFNs), causes cytokine storm, which is a major COVID-19 factor that potentially leads to fatal outcomes.^31^ INFs, particularly INF-α and IFN-β activate other cytokines such as IL-12 and the type II interferon cytokine, IFN-γ.^31^ Although Type I IFN signalling cascades constrict inflammation caused by the virus, cytokines such as IL-10 block the type-I IFN response.^32^ However, in severe infections with SARS-CoV-2, the type-I IFN signalling is abnormal, leading up to altered development of adaptive immunity.^31^ Substantial changes in galactosylation levels in severe COVID-19 infection resulting in a higher abundance of agalactosylated IgG molecules, compared to mild COVID-19, are related to the proinflammatory effects of IgG.^33^ This proinflammatory function of agalactosylation on one hand acts through complement system activation.^34^^,^^35^ On the other hand, since galactose is a prerequisite for sialylation, agalactosylation also has an indirect proinflammatory effect. Previous studies reported decreased galactosylation both in anti-S IgG1 and total IgG1 in severe COVID-19 compared to mild,^28^ which is consistent with changes in total IgG glycome. Decreased sialylation in total IgG glycome was associated with a disease severity. A similar observation was reported in other studies, where severe COVID-19 was characterized by lower anti-S IgG1 sialylation compared to mild COVID-19.^13^ On the other hand, Pongracz and colleagues reported that elevated sialylation levels on anti-S IgG1 were associated with increased disease severity, again supporting the hypothesis of very dynamic changes in IgG glycosylation that happen during severe COVID-19 on the level of antigen-specific and total IgG, like discussed above.^28^ While sialylation has been studied as a critical feature in anti-inflammatory activity, its role as an anti-inflammatory feature in COVID-19 needs to be further explored as well as the question of cause and consequence of glycosylation changes during COVID-19 in relation to inflammation.^36^

In this study, we observe multiple statistically significant changes in IgG glycome composition in severe COVID-19 patients during the course of illness. The most statistically significant changes included decreasing levels of bisecting GlcNAc, increasing levels of agalactosylated glycans and decreased sialylation during illness. This indicates that a more proinflammatory change of IgG glycome may be associated with an increased risk for severe COVID-19 and suggests that inter-individual differences in IgG glycosylation and their changes during the disease should be studied in more detail.

### Caveats and limitations

Our study benefits from a large sample size and the longitudinal nature of our data and, to our knowledge, is the first study to investigate longitudinal changes in total IgG glycome in both mild and severe SARS-CoV-2 patients. Nevertheless, our study also has some limitations. First, samples were collected as the sets of convenience comprising no healthy controls, with mild cases collected in the UK and BiH cohorts, while severe cases were collected in two HR cohorts (HR1 in the second wave and HR2 in the third wave of the pandemic), resulting in sample sets with unequal sex and age distributions. Second, detailed biochemical data wasn't available for all study participants and therefore wasn't included in the analysis. We aim to address this aspect in our future studies. In addition, we are aware that recruiting patients in hospital centres from different countries may introduce biases in sample collection and the genetic and geographical background of patients between cohorts. However, these were the major centres for the care of COVID -19 patients, so we consider them to be reasonably representative of the COVID -19 pandemics on a global scale.

## Contributors

G.L. conceptualized the study. B.P., B.J.O., A.M.V., I.H. lead the clinical teams providing the samples and co-morbidities stratification, T.B, D.M., A.M.V., B.J.O., A.V., L.Đ., I.J. obtained the primary samples; T.B., D.M., I.H., L.Đ., I.J. processed the samples. T.P. and I.T.A. performed glycan analysis. F.V. conducted statistical analysis. F.V., I.T.A., and T.P. produced the figures. F.V., I.T.A., G.L., and T.P. interpreted the results. T.P. wrote the first draft of the manuscript. G.L., I.T.A., F.V., A.M., I.L. critically revised the initial draft of the manuscript*.* I.T.A helped to edit the manuscript. All authors reviewed and approved the final version of the manuscript.

## Data sharing statement

In this study, we used deidentified (codes instead of names) data about each individual participant with COVID-19. Depending on the aim of proposed research, the institution that holds data will consider each data access request on a case-by-case basis and decide whether to share data with the requester or not. Regarding the Nottingham cohort from United Kingdom, you can contact Prof Ana M. Valdes from School of Medicine, University of Nottingham, Nottingham (ana.valdes@nottingham.ac.uk). Regarding the BiH cohort from Bosna and Hercegovina you can contact Prof Tamer Bago (tamer.bago@gmail.com) and regarding HR cohort from the University Hospital Dubrava, Croatia please contact, Prof Irzal Hadžibegović (irzalh@gmail.com). Glycan data generated during this study are available from the corresponding author (glauc@genos.hr), immediately following publication, upon reasonable request.

## Declaration of interests

G.L. is the founder and owner of Genos Ltd, a private research organization that specializes in high-throughput glycomic analysis and has several patents in this field. T.P., F.V. and I.T.A. are employees of Genos Ltd. Other authors declare no competing interests.

## Appendix Supplementary materials

Image, application 1
