# Supplementary material for: IgG N-glycome changes during the course of severe COVID-19: An observational study
Source: eBioMedicine. 2022 Jun 27;81:104101. doi: 10.1016/j.ebiom.2022.104101 (PMC9234382; doi:10.1016/j.ebiom.2022.104101)
Supplement: Supplementary file 1 [file mmc1.docx]

**Supplementary Table 1.** Flow chart of disposition of patients through the study.


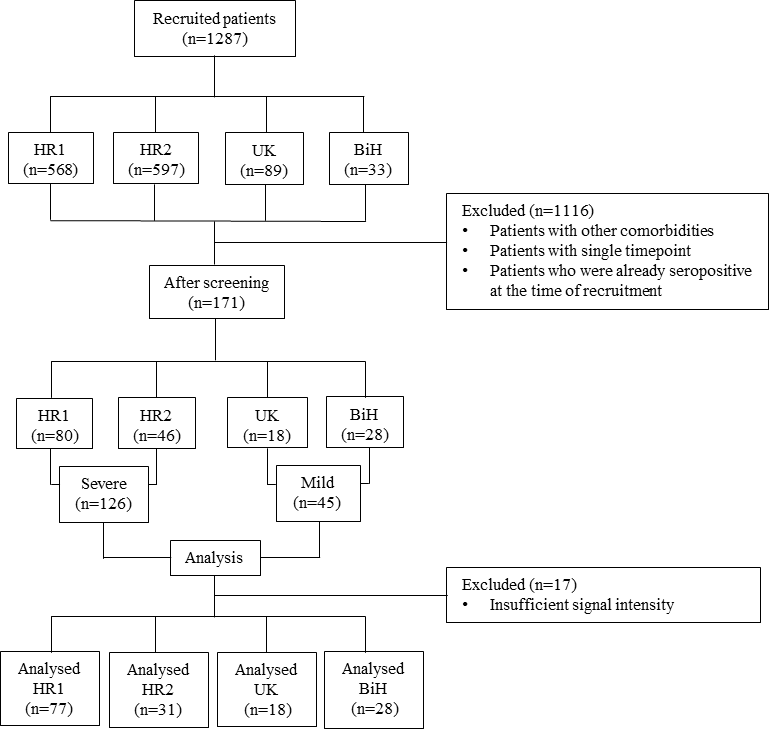


**Supplementary Table 2.** Composition of the IgG glycome. All N-glycans consist of a core sugar sequence of two *N*-acetylglucosamines (GlcNAc) and three mannose residues, as previously described by Pučić and colleagues (2011.). F indicates a core fucose, α1–6 linked to the inner GlcNAc. A1 represents one GlcNAc antenna on trimannosyl core. A2 is a biantennary glycan with both GlcNAcs as β1–2 linked. B represents a bisecting GlcNAc linked β1–4 to the β1–3 mannose. G represents a galactose which is β1–4 linked to the antenna: [3]G1 and [6]G1 indicate that the galactose is either on the antenna of the α1–3 or α1–6 mannose, respectively. S1 and S2 are molecules of sialic acids linked to antennary galactose. Structural schemes are given in terms of GlcNAc (blue square), mannose (green circle), fucose (red triangle), galactose (yellow circle) and sialic acid (pink romb).

1. IgG glycome labeled with RapiFluor-MS was separated into 22 chromatographic peaks by HILIC-UHPLC-FLR.
2. IgG glycome labeled with InstantPC was separated into 25 chromatographic peaks by HILIC-UHPLC-FLR.

| **RapiFluor-MS labeled glycans** | | | **InstantPC labeled glycans** | | |
| --- | --- | --- | --- | --- | --- |
| Glycan peak | Peak composition | Structure | Glycan peak | Peak composition | Structure |
| RF - GP1 | FA1 | 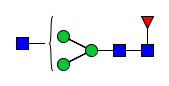 |  |  |  |
| RF - GP2 | A2 | 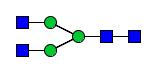 | IPC - GP1 | A2 | 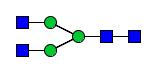 |
| RF - GP3 | FA2 | 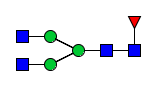 | IPC – GP2 | FA2 | 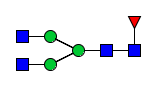 |
|  | A2B (minor) | 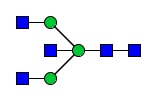 | IPC – GP3 | FA2B | 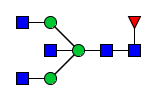 |
| RF – GP4 | FA2B | 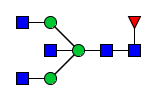 | RF – GP4 | A2[6]G1 | 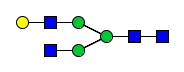 |
|  | FA1G1 (minor) | 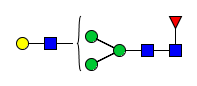 | IPC – GP5 | A2[3]G1 | 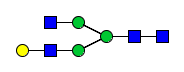 |
| RF – GP5 | A2[6]G1 | 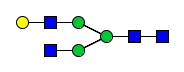 | IPC – GP6 | FA2[6]G1 | 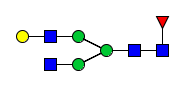 |
| RF – GP6 | A2[3]G1 | 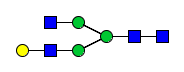 | IPC - GP7 | FA2[3]G1 | 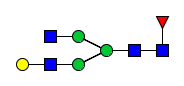 |
| RF – GP7 | FA2[6]G1 | 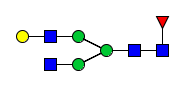 | IPC - GP8 | FA2[6]BG1 | 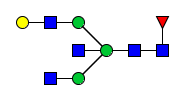 |
| RF – GP8 | FA2[3]G1 | 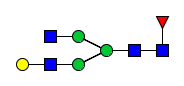 | IPC - GP9 | FA2[3]BG1 | 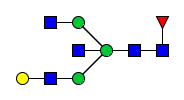 |
| RF – GP9 | FA2[6]BG1 | 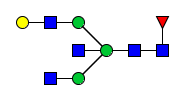 | IPC - GP10 | A2G2 | 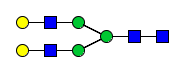 |
| RF - GP10 | FA2[3]BG1 | 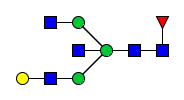 | IPC - GP11 | A2BG2 | 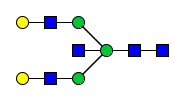 |
| RF - GP11 | A2G2 | 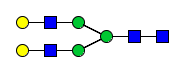 | IPC - GP12 | FA2G2 | 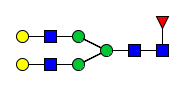 |
| RF - GP12 | FA2G2 | 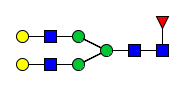 | IPC - GP13 | FA2BG2 | 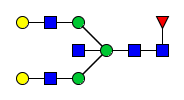 |
|  | A2BG2 (minor) | 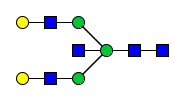 | IPC - GP14 | FA2G1S1 | 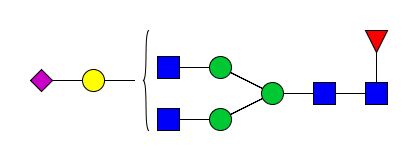 |
| RF - GP13 | FA2BG2 | 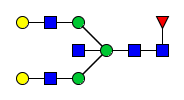 | IPC - GP15 | FA2G1S1 | 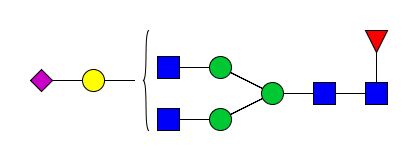 |
| RF - GP14 | FA2G1S1 | 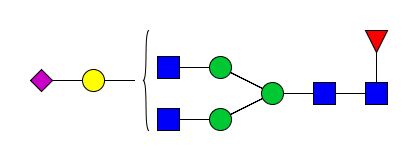 | IPC - GP16 | FA2BG1S1 | 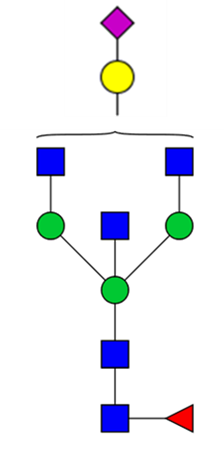 |
| RF - GP15 | A2G2S1 | 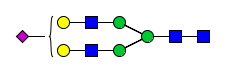 | IPC - GP17 | FA2BG1S1 | 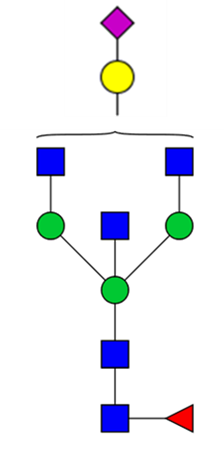 |
| RF - GP16 | FA2G2S1 | 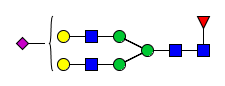 | IPC - GP18 | A2G2S1 | 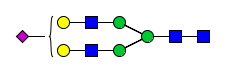 |
| RF - GP17 | FA2BG2S1 | **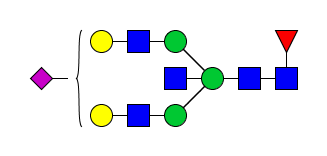** | IPC - GP19 | FA2G2S1 | 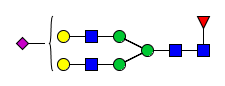 |
| RF – GP18 | not determined |  | IPC – GP20 | FA2BG2S1 | **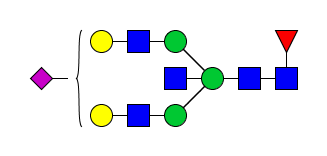** |
| RF – GP19 | A2G2S2 | 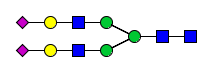 | IPC - GP21 | not determined |  |
| RF - GP20 | A2BG2S2 | 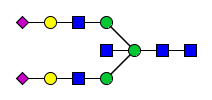 | IPC - GP22 | A2G2S2 | 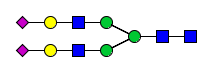 |
| RF - GP21 | FA2G2S2 | 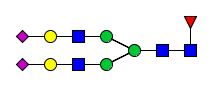 | IPC - GP23 | A2BG2S2 | 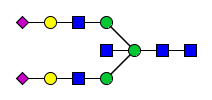 |
| RF - GP22 | FA2BG2S2 | 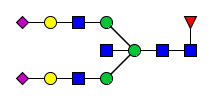 | IPC - GP24 | FA2G2S2 | 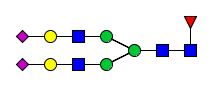 |
|  |  |  | IPC - GP25 | FA2BG2S2 | 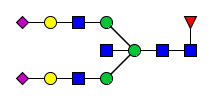 |

**Supplementary Table 3.** IgG derived glycan traits calculated out of:

1. 22 directly measured IgG glycan peaks labeled with RapiFluor-MS
2. 25 directly measured IgG glycan peaks labeled with InstantPC

G0 – agalactosylated N-glycans, G1 – N-glycans with one galactose, G2 – N-glycans with two galactoses, S – sialylated N-glycans, B – N-glycans with bisecting GlcNAc, F – N-glycans with core fucose.

| **Derived trials** | **RapiFluor-MS peaks formula** | **InstantPC peaks formula** |
| --- | --- | --- |
| **G0** | RF-GP1 + RF-GP2 + RF-GP3 + RF-GP4 | IPC-GP1 + IPC-GP2 + IPC-GP3 |
| **G1** | RF-GP5 + RF-GP6 + RF-GP7 + RF-GP8 + RF-GP9 + RF-GP10 | IPC-GP4 +IPC-GP5 + IPC-GP6 + IPC-GP7 + IPC-GP8 + IPC-GP9 |
| **G2** | RF-GP11 + RF-GP12 + R-FGP13 | IPC-GP10 + IPC-GP11 + IPC-GP12 + IPC-GP13 |
| **B** | RF-GP4 + RF-GP9 + RF-GP10 + RF-GP13 + RF-GP17 + RF-GP20 + RF-GP22 | IPC-GP3 + IPC-GP8 + IPC-GP9 + IPC-GP11 + IPC-GP13 + IPC-GP16 + IPC-GP17 + IPC-GP20 +IPC-GP23 + IPC-GP25 |
| **F** | RF-GP1 + RF-GP3 + RF-GP4 + RF-GP7 + RF-GP8 + RF-GP9 + RF-GP10 + RF-GP12 + RF-GP13 + RF-GP14 + RF-GP16 + RF-GP17 + RF-GP21 + RF-GP22 | IPC-GP2 + IPC-GP3 + IPC-GP6 + IPC-GP7 + IPC-GP8 + IPC-GP9 + IPC-GP12 + IPC-GP13 + IPC-GP14 + IPC-GP15 + IPC-GP16 + IPC-GP17 + IPC-GP19 +IPC-GP20 + IPC-GP24 + IPC-GP25 |
| **S** | RF-GP14 + RF-GP15 + RF-GP16 + RF-GP17 + RF-GP19 + RF-GP20 + RF-GP21 + RF-GP22 | IPC-GP14 + IPC-GP15 + IPC-GP16 + IPC-GP17 + IPC-GP18 + IPC-GP19 + IPC-GP20 + IPC-GP22 + IPC-GP23 + IPC-GP24 + IPC-GP25 |


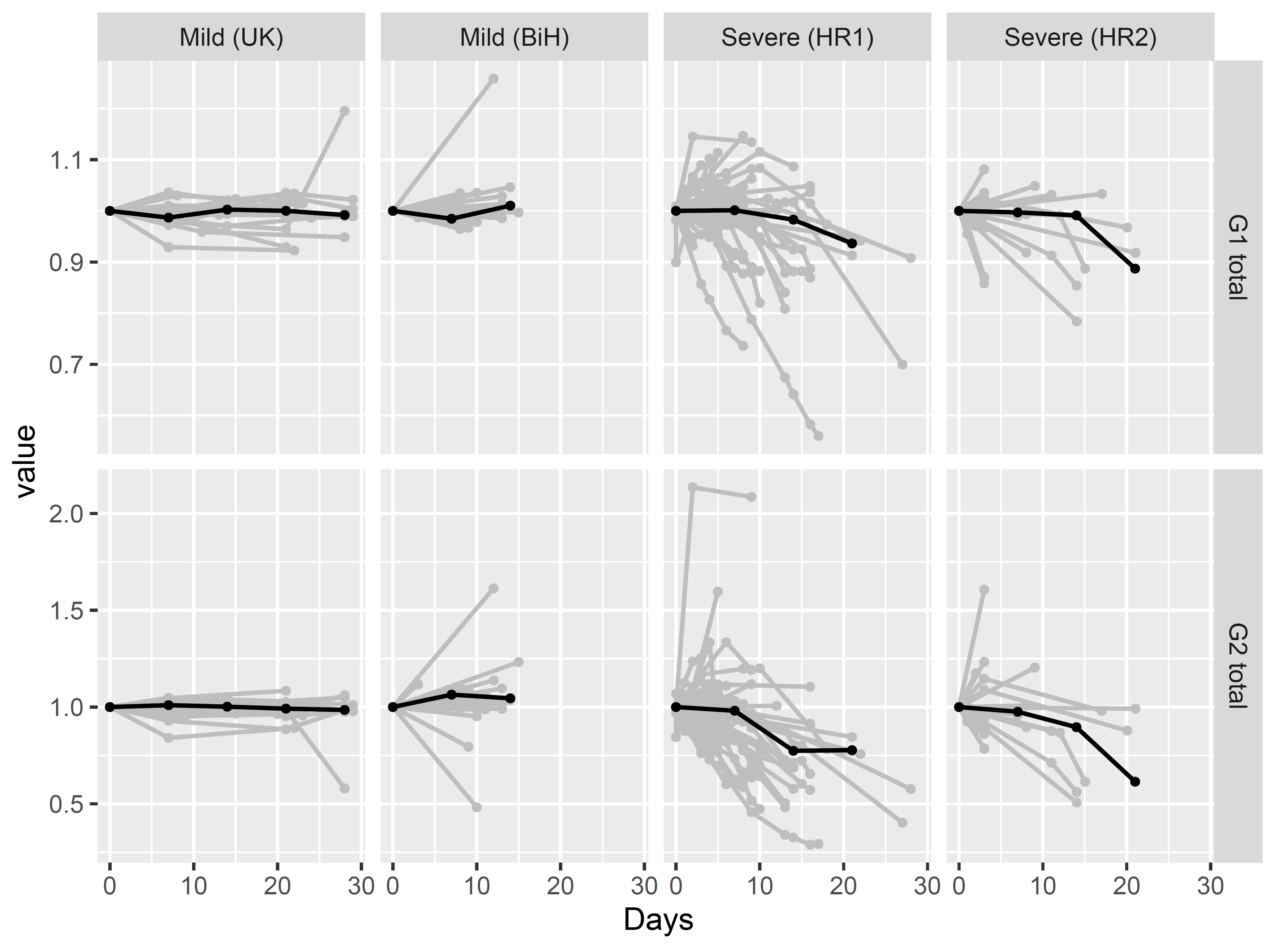


**Supplementary Figure 1. Alternation in IgG glycan composition in mild (UK, n= 18; BiH, n=28) and severe (HR1, n=77; HR2, n=31) COVID-19.** Standardised glycan measurements are represented on the y-axis, while time in days is presented on the x-axis. Black dots represent 7-day, cohort-specific averages of standardized glycan measurements. G1 – N-glycans with one galactose, G2 – N-glycans with two galactoses; x - Time (days); y - Standardized Glycan Measurement. Follow-up data for 30 days is presented. Additional information is available in Table 3.
